# Protect the recurrent laryngeal nerves in US-guided microwave ablation of thyroid nodules at Zuckerkandl tubercle: a pilot study

**DOI:** 10.1186/s12885-024-12020-3

**Published:** 2024-02-26

**Authors:** Ziyue Hu, Lu Wang, Man Lu, Wei Yang, Xiaobo Wu, Jinshun Xu, Min Zhuang, Shishi Wang

**Affiliations:** Department of Ultrasound, Sichuan Clinical Research Center for Cancer, Sichuan Cancer Hospital & Institute, Sichuan Cancer Center, Affiliated Cancer Hospital of University of Electronic Science and Technology of China, Chengdu, China

**Keywords:** Recurrent laryngeal nerves, Microwave ablation, Thyroid nodules at Zuckerkandl tubercle

## Abstract

**Background:**

To evaluate the safety and efficacy of US-guided microwave ablation in patients with thyroid nodules at Zuckerkandl tubercle.

**Methods:**

103 consecutive patients with thyroid nodules at Zuckerkandl tubercle (ZTTN) were enrolled in this study from November 2017 to August 2021. Prior to the surgery or US-guided microwave ablation (MWA), preoperative ultrasound visualization of the recurrent laryngeal nerve (RLN) and ZTTN was performed, the size and the position relationship between them were observed. Patients were followed up at 1, 3, 6, and 12 months after MWA and the volume reduction rates (VRR) of the thyroid nodules were analyzed.

**Results:**

All patients successfully had the RLN and ZTTN detected using ultrasound before surgery or ablation with a detection rate of 100%. For the 103 patients, the majority of ZTTN grades were categorized as grade 2, with the distance from the farthest outside of ZTTN to the outer edge of thyroid ranging between 6.0 and 10.0 mm. The position relationship between ZTTN and RLN was predominantly type A in 98 cases, with type D observed in 5 cases. After MWA, the median nodule volume had significantly decreased from 4.61 (2.34, 8.70) ml to 0.42 (0.15, 1.41) ml and the VRR achieved 84.36 ± 13.87% at 12 months. No nodules regrew throughout the 12-month follow-up period. Of the 11 patients experienced hoarseness due to RLN entrapment before ablation, 7 recovered immediately after separation of the RLN and ZTTN during MWA, 2 recovered after one week, and the other 2 recovered after two months.

**Conclusions:**

The RLN is closely related to ZTTN and mainly located at the back of ZTTN. The RLN can be separated from ZTTN by hydrodissection during MWA. US-guided MWA is a safe and effective treatment for ZTTN.

## Background

**Fig. 1:**
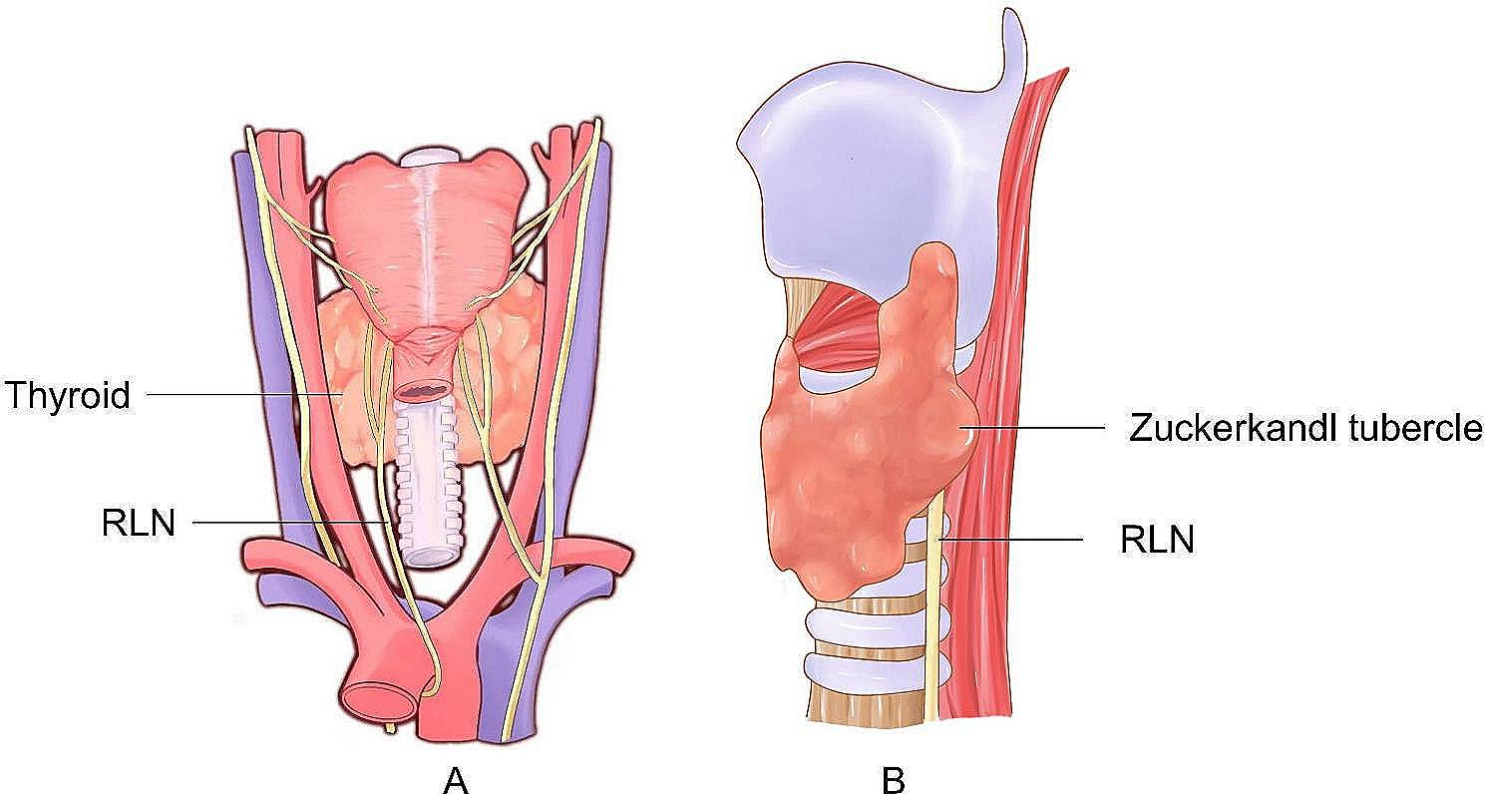
(**A**) Recurrent laryngeal nerve (RLN) anatomy and (**B**) the relationship of Zuckerkandl tubercle with the RLN

The incidence of thyroid nodules is on the rise annually, and it is estimated that 20-76% of adults have one or more thyroid nodules [1, 2]. With the development of image-guided approaches for the treatment of thyroid nodules, a growing number of patients and clinicians have favored minimally invasive approaches, including percutaneous laser ablation (LA), radiofrequency ablation (RFA), and Microwave ablation (MWA) in recent years [3–7]. MWA is a new and important minimally invasive treatment option that can be used to treat benign and malignant tumors of the thyroid, breast, liver, and lung [8–12]. Numerous studies have demonstrated that MWA is a safe and effective technique to reduce the volume of thyroid nodules [5, 13]. MWA offers many advantages, such as a larger ablation volume, higher ablation speed, less impact on nearby blood vessels, and high specificity [14–17]. However, recurrent laryngeal nerve (RLN) injury is still a prevalent complication, with neurological abnormalities described in 1.0–9.1% of patients [17–19]. Moreover, if the patient has thyroid nodules at the Zuckerkandl tubercle (ZTTN), the RLN is at a higher risk of damage as the ZTTN is located in close proximity to the RLN (see Fig. 1). Injury to the RLN can lead to dysphagia and vocal hoarseness, while bilateral RLN injury will result in bilateral vocal cord paralysis and may even cause breathing difficulties, which could be life-threatening. Thus, it is worth researching ways to avoid RLN injury during the MWA. In this context, we employed high-frequency ultrasound (US) to preoperatively visualize the RLN and to lower the risk of the RLN injury during MWA for ZTTN. The purpose of our study was to evaluate the safety and efficacy of US-guided MWA in patients with ZTTN.

## Materials and methods

### Patient population

From November 2017 to August 2021, 10 patients with ZTTN combined with RLN invasion who were planning to have a surgery were first included. Then 103 consecutive patients with ZTTN were enrolled in this study. For those 103 patients, the inclusion criteria were as follows: (1) presence of ZTTN; (2) documented pressure symptoms and/or aesthetic issues; (3) confirmation of benign thyroid nodules with two separate fine-needle aspirations (FNA) cytology; (4) routine blood work, blood biochemistry, coagulation function, and serum thyroid hormone and thyrotropin levels within the normal ranges; (5) normal movement of bilateral vocal cords confirmed by preoperative trans-laryngeal ultrasonography; and (6) patients refusal of surgery and choice of MWA treatment. The exclusion criteria were: (1) pregnancy, profound coagulopathy, and severe systemic diseases that contraindicated ablation treatment.; (2) previous thyroid, parathyroid, or neck dissection surgery or MWA treatment; and (3) thyroid nodules that were difficult to monitor during MWA, such as retrosternal thyroid nodules. Informed consent was obtained from all patients, and the Medical Ethics Committee approved this study at Sichuan Cancer Hospital and Institute (SCCHEC-03-2017-008).

### Voice evaluation

Two laryngologists evaluated the voice using the GRBAS scale. This perceptual rating scale considers various factors, such as overall grade, roughness, breathiness, asthenia, and strain of the voice. The listeners assessed the speech samples based on overall quality, roughness, and breathiness, and rated each one on a scale of 0 (normal), 1 (mild), 2 (moderate), and 3 (severe). The ratings from both listeners were averaged to determine the final values.

### US-guided MWA procedure

The US-guided MWA procedure for thyroid nodules was performed by radiologists with over three years of experience in MWA. The KY-2000 MWA instrument (Kangyou Medical, Nanjing, China), whose generator can generate power of up to 1-100 W at 2450 MHz, was used to administer microwave energy. Ultrasound and Contrast-Enhanced Ultrasound (CEUS) were performed using Philips EPIQ7 (Philips Healthcare, Bothell, WA, USA) ultrasonic diagnostic instrument with a 5–12 MHz linear matrix transducer.

**Fig. 2:**
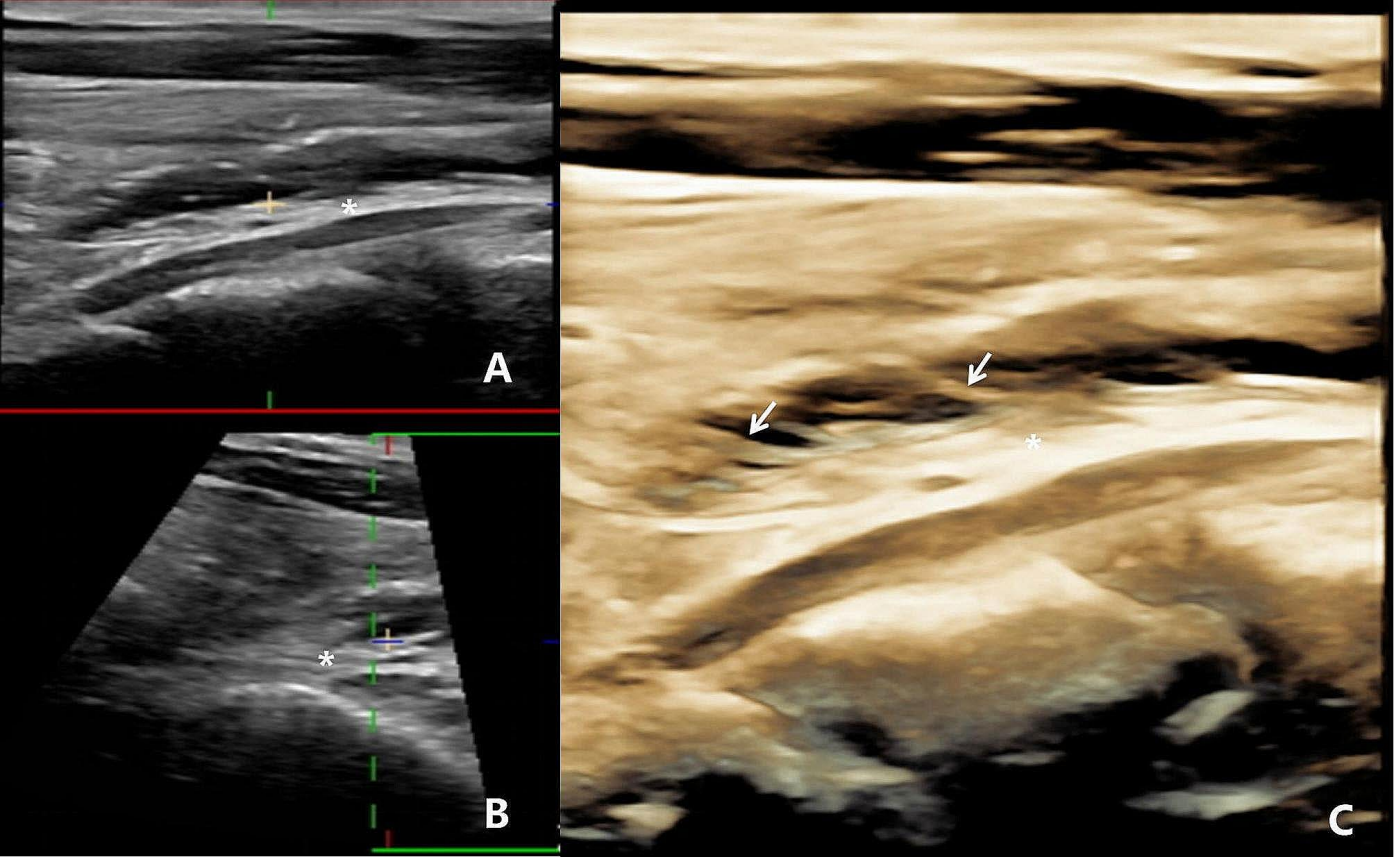
Administration of physiological saline solution between the thyroid capsule and the RLN. (**A**) Long axial section showing RLN (asterisk); (**B**) Short axial section showing the RLN (asterisk); (**C**) Three-dimensional ultrasound imaging showing numerous small branches of the RLN (asterisk) (white arrow)

The patient was placed in a supine position with the neck fully exposed and given local anesthetic at the puncture site. The thyroid and subcutaneous tissues were dissected using 10 ml of 2% lidocaine. Under US guidance, a physiological saline solution was administered to create a liquid barrier between the thyroid capsule and adjacent critical structures to prevent thermal injury (Fig. 2). Under US guidance, a physiological saline solution was administered to create a liquid barrier between the thyroid capsule and adjacent critical structures to prevent thermal injury. At this point, the RLNs were clearly visible, identified as hypoechoic oval shapes with surrounding hyperechoic tissue using color Doppler mode, which distinguishes them from microscopic blood arteries. The RLN was tracked upwards to its entry point in the laryngeal area and then downwards to the supraclavicular area. The width and thickness of the RLN, as well as the distance between the RLN and ZTTN, were measured and recorded. Additionally, the height of ZTTN was recorded, which is defined as the distance from the farthest outside of the ZTTN to the outer edge of the thyroid. Based on its height, ZTTN was classified into four categories: 0 (unrecognizable), 1 (ZTTN height not more than 5.0 mm), 2 (ZTTN height between 6.0 and 10.0 mm) and 3 (ZTTN height exceeding 10.0 mm) [20]. The position relationship between ZTTN and RLN was classified into four types: Type A (RLN located at the back of ZTTN), Type B (RLN located at the front of ZTTN), Type C (RLN passes through the substance of ZTTN) and Type D (RLN located at the side of ZTTN) [21].

Then, an ablation antenna was positioned under US guidance through a small 2 mm incision of the skin at the percutaneous site. The power output of the 30 W moving shot technique, leverage, and pry-off method was applied during MWA. If cystic components were detected, fluid was aspirated with a fine needle, and the capsule was rinsed repeatedly with physiological saline solution before ablation. Then ablation was terminated when the hyperechoic zone completely covered all the treated portion of the thyroid nodule. The patient’s voice and vocal cords were monitored during the operation, and the operation was stopped if dysphonia or vocal cord hemiplegia occurred.

CEUS was conducted about 30 min after MWA to avoid steam interference. SonoVue (Bracco Suisse SA, Italy) was used as the ultrasound contrast agent. It was dissolved in 5 ml of saline before being immediately injected as a bolus into the antecubital vein, followed by a 5 ml saline flush. If the CEUS revealed no enhancement in the nodules in numerous orientations and slices, it proved that the thyroid nodules were completely ablated.

### Follow-up evaluation

After the ablation procedure above, all patients underwent laryngeal ultrasonography within 30 min, and those who exhibited aberrant vocal cord activity were suspected to have RLN injury. Maximal nodule diameters and nodule volumes were measured at 1, 3, 6, and 12 months post-ablation. Clinical outcomes were monitored, including percentage reduction in volume, GRBAS scale, and sequelae. The volume reduction ratio (VRR) was calculated using the following formula: VRR = (initial volume-final volume)×100/initial volume.

### Surgical procedure for patients with RLN invasion

During this study, 10 patients were identified with ZTTN combined with RLN invasion on preoperative ultrasound, and pathological examination confirmed that they had papillary thyroid carcinoma. All patients underwent total thyroidectomy and standard central lymph node dissection, and those with lateral cervical lymph node metastasis underwent neck lymph node dissection. For cases where the tumor slightly invaded or adhered to the RLN, the RLN was carefully peeled off with a surgical magnifying glass. However, when vocal cord paralysis (VCP) was present before surgery, or when there was extensive infiltration, nerves were usually sacrificed. In such cases, intraoperative nerve monitoring (IONM) was performed. It should also be noted that when bilateral RLN injury was confirmed, tracheotomy was typically performed during the operation.

### Statistical analysis

SPSS 22.0 (SPSS Inc., Chicago, IL, USA) was used for statistical analysis. The mean ± standard deviation (SD) was used to characterize measurement data, including nodule size, patient age, the GRBAS scale, and the width and thickness of the RLN. Medians and quartiles were used to describe nodule volume. The non-parametric Wilcoxon signed-rank test was applied for pairwise comparison between pre-ablation and post-ablation nodule volumes at each follow-up time point. Statistical significance was defined as *p* < 0.05.

## Results

### Ultrasound observation confirmation on patients with RLN invasion

**Fig. 3:**
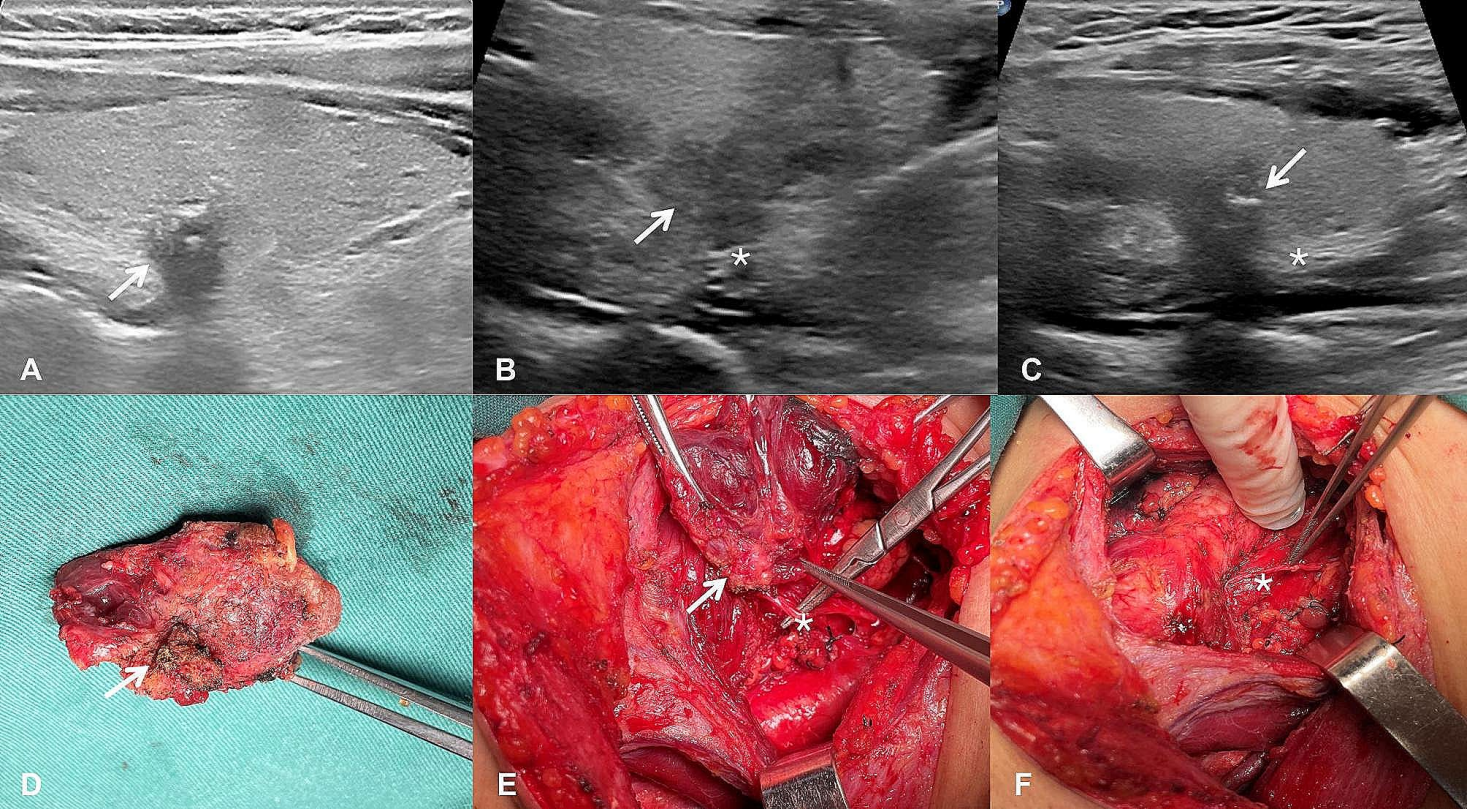
A 26-year-old male patient with tubercle of Zuckerkandl combined with thyroid nodules (ZTTN, white arrow) in the right lobe underwent underwent total thyroidectomy and standard central lymph node dissection. The pathological results of the patient confirmed that were papillary thyroid carcinoma. (**A**) The distance from the farthest outside of ZTTN (white arrow) to the outer edge of thyroid is 6.0 mm (ZTTN grade 2). (**B**) The recurrent laryngeal nerve (RLN, asterisk) is located at the back of ZTTN (white arrow) and the relationship between ZTTN and RLN is Type A. (**C**) The ZTTN (white arrow) invade the RLN(asterisk) and the boundary between ZTTN and RLN is unclear. (**D**) Gross specimen of right lobe of thyroid gland and ZTTN (white arrow). (**E**) Intraoperative findings demonstrated the RLN (asterisk) is located at the back of ZTTN (white arrow) and the ZTTN invade the RLN. (**F**) The separated RLN (asterisk) is very fine after right thyroidectomy

In this study, 10 patients with RLN invasion were included. During the operation, 6 patients were found to have adhesion between the RLNs and ZTTN, while the RLNs of the other 4 patients were directly invaded by the tumor. Among these ten patients, 3 had a ZTTN grade of 1, 6 had a grade of 2, and 1 had a grade of 3. The relationship between the recurrent laryngeal nerve and ZTTN was type A in 8 cases and type D in 2 cases. The results of ZTTN grading were consistent with the findings of RLN and ZTTN during the operation (Fig. 3).

### Clinical characteristics of the study population with US-guided MWA

**Fig. 4:**
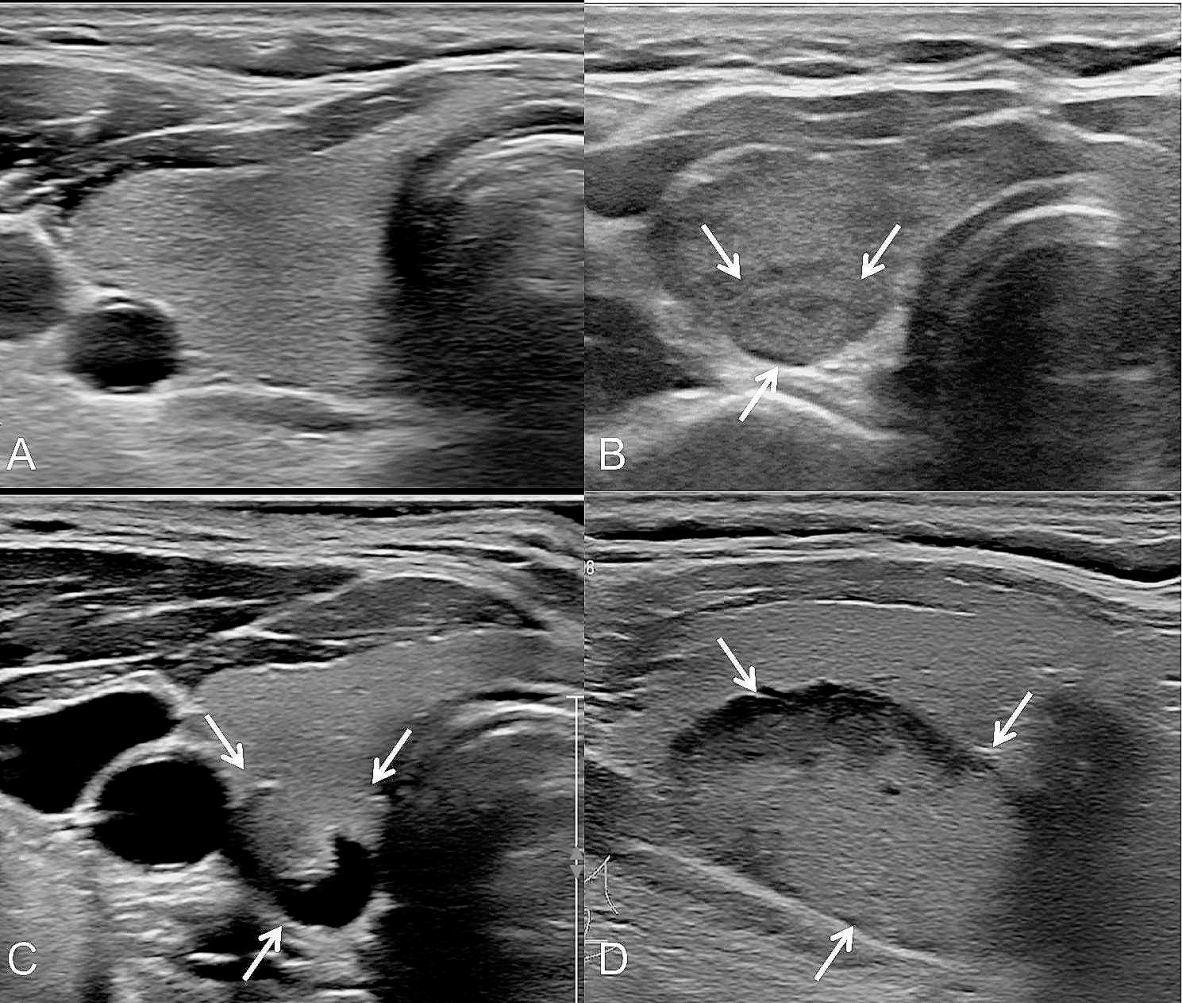
Different types of ZTTN. (**A**) shows the ZTTN grade 0 (unrecognizable) in the short-axis section of thyroid. (**B**) shows that solid ZTTN (white arrow) in the short-axis section with the distance from the farthest outside of ZTTNs (white arrow) to the outer edge of the thyroid not exceeding 5.0 mm (ZTTN grade 1). (**C**) shows that the location of cystic-solid ZTTN (white arrow) in the short-axis section with the distance from the farthest outside of the ZTTNs (white arrow) to the outer edge of thyroid being 7.0 mm (ZTTN grade 2). (**D**) shows that the location of solid ZTTN (white arrow) in long-axis section with the distance from the farthest outside of the ZTTNs (white arrow) to the outer edge of thyroid exceeding 10.0 mm (ZTTN grade 3)

A total of 103 patients with ZTTN underwent US-guided MWA (see Fig. 4). All patients had ZTTN that were closely related to the RLN based on preoperative ultrasound evaluation. The baseline characteristics of the thyroid nodules are summarized in Table 1. Of the 103 patients, 11 were male and 92 were female, with ages ranging from 19 to 80 years (mean age: 43.7 ± 12.4 years). The mean diameter and mean volume of ZTTN were 27.60 ± 8.91 mm and 6.23 ± 5.82 ml, respectively. For the fluid component assessment, 24 (23.3%) nodules were solid (≤ 10% fluid component), 55 (53.4%) were predominantly solid (11–50% fluid component), and 24 (23.3%) were predominantly cystic nodules (51–90% fluid component).

**Table 1:** Baseline characteristics of patients with ZTTN

Patients	*n* = 103
Age(years)	43.7 ± 12.4 [19–80]
Sex	
Male	11
Female	92
ZTTN	
Mean diameter	27.60 ± 8.91 mm
Mean volume	6.23 ± 5.82 ml
Nodule composition	
Solid	*n* = 24
Mixed	*n* = 55
Mainly cystic	*n* = 24
Location	
Left lobe	*n* = 36
Right lobe	*n* = 67

### Ultrasound visualization results of RLN and ZTTN

All patients successfully had the RLN detected before ablation with a detection rate of 100%. The position relationship between ZTTN and RLN could also be clearly seen. Among them, 76 patients had RLN that can be separated from ZTTN, while 27 patients found partial adhesion between RLN and ZTTN. During MWA, RLN were separated from ZTTN after hydrodissection with a distance greater than 5 mm in 99 patients. The remaining 4 patients had severe adhesion where hydrodissection could not completely separate the RLN and ZTTN and required a needle to be used for separation under ultrasound guidance. The width and thickness of the RLN before ablation were found as 2.768 ± 0.399 mm and 2.510 ± 0.349 mm, respectively (see Table 2).

It was found that ZTTN grade 2 was the most prevalent in this study, followed by ZTTN grade 3 with 31 patients and ZTTN grade 1 with only 3 patients. The majority of cases (98) had a type A relationship between ZTTN and RLN, while only 5 cases were type D. Specifically, when ZTTN was grade 1, the relationship between ZTTN and RLN was 33.3% type A, 0% type B, 0% type C and 66.7% type D. When ZTTN was grade 2, the relationship between ZTTN and RLN was 97.1% type A, 0% type B, 0% type C, and 2.9% type D. For ZTTN grade 3, 96.8% of cases had a type A relationship between ZTTN and RLN, 0% had type B, 0% had type C, and 3.2% had type D (see Table 3).

**Table 2:** The measurements of parameters associated with RLN

	Mean ± SD(mm)	Range
The width of the RLN	2.768 ± 0.399	2.1–3.7
The thickness of the RLN	2.510 ± 0.349	2.0–3.0

**Table 3:** ZTTN grade and the relationship between ZTTN and RLN

ZTTN grade	The relationship between ZTTN and RLN			
	type A	type B	type C	type D
0	0	0	0	0
1	1	0	0	2
2	67	0	0	2
3	30	0	0	1

**Fig. 5:**
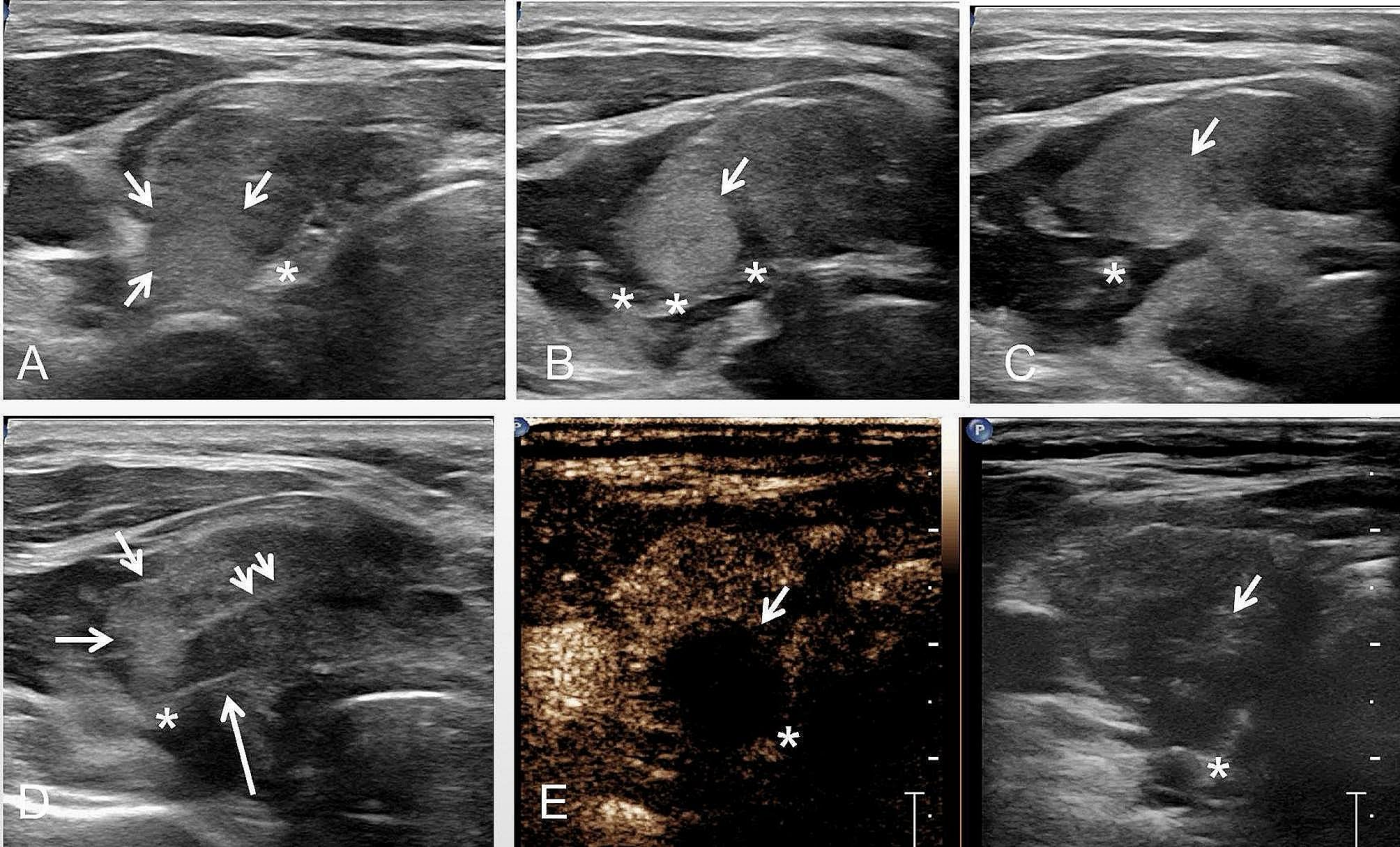
A 45-year-old female patient with nodular goiter in the left lobe of the thyroid underwent US-guided MWA. (**A**) The relationship between the RLN (asterisk) and the ZTTN (white arrow). The distance from the farthest outside of ZTTNs (white arrow) to the outer edge of thyroid is 4.0 mm (ZTTN grade 1). The RLN (asterisk) is located at the side of ZTTN (white arrow), and the relationship between ZTTN and RLN is Type D. (**B**) shows the relationship between the ZTTN (white arrow) with the RLN (asterisk) in the long axial section after the hydrodissection. (**C**) After the physiological saline solution was injected, the distance between the RLN and ZTTN significantly widened in the short-axis section. (**D**) The ablation antenna (double short white arrow) was placed inside the ZTTN (white arrow), and the injection needle (long white arrow) was placed between the ZTTN (white arrow) and RLN (asterisk). During the microwave ablation, the physiological saline solution could be continuously injected between the ZTTN (white arrow) and RLN (asterisk) to protect the RLN. (**E**) After MWA, the contrast-enhanced ultrasonography showed no enhancement in arterial and venous phase of ZTTN (white arrow), but high enhancement in RLN (asterisk), suggesting that RLN was not damaged during ablation

**Fig. 6:**
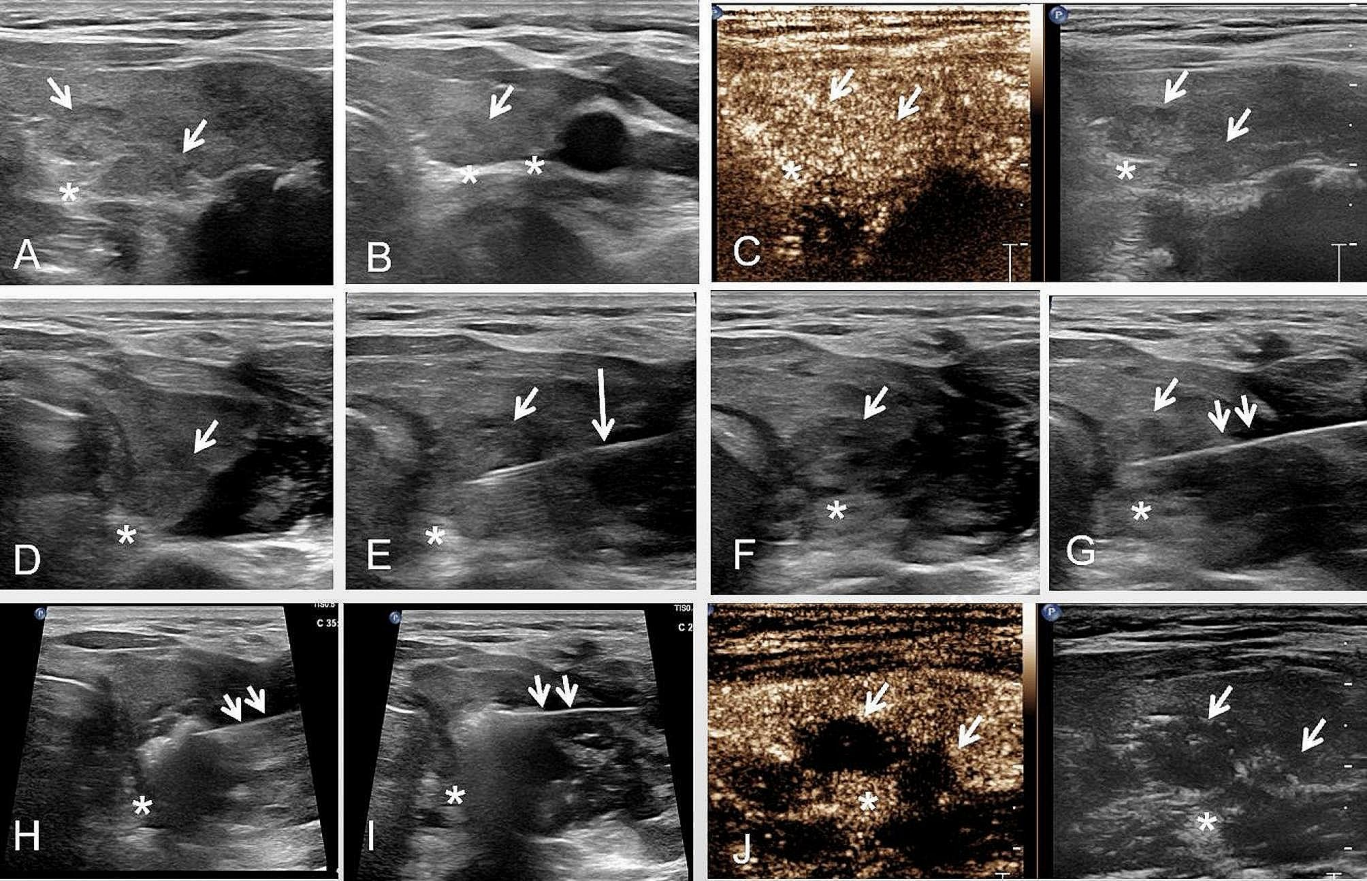
A 50-year-old female patient with ZTTN (white arrow) in the right lobe underwent US-guided MWA. The surgeon was on the side of the patient’s head during the procedure. (**A**) Two ZTTNs (white arrow) were closely related to the RLN (asterisk) in the short axial section. The distance from the farthest outside of ZTTNs (white arrow) to the outer edge of thyroid is 5.0 mm (ZTTN grade 2). (**B**) In the long axial section, one of the ZTTN (white arrow) was observed closely related to the RLN (asterisk). The RLN (asterisk) was located at the back of the ZTTN (white arrow), and the relationship between ZTTN and RLN was Type A. (**C**) The contrast-enhanced ultrasound showed high enhancement of ZTTN (white arrow) and RLN (asterisk). (**D**) After the hydrodissection began, the ZTTN (white arrow) could be seen to separate from the RLN (asterisk). (**E**) To ablate the superficial ZTTN, the injection needle (long white arrow) was placed between the ZTTN (white arrow) and the RLN (asterisk). (**F**) After continuous injection of physiological saline solution, the distance between the RLN (asterisk) and ZTTN (white arrow) was significantly widened. (**G**) The ablation antenna (double white arrows) was inserted into the superficial ZTTN (white arrow). (**H**-**I**) During MWA, the ablation antenna (double white arrows) was tilted upward to increase the distance between ZTTN and the RLN (asterisk). (**J**) Contrast-enhanced ultrasound after MWA showed that the ZTTN (white arrow) was not enhanced, indicating that the ZTTN was completely ablated. The RLN (asterisk) was highly enhanced, and the patient’s voice did not change significantly after MWA, indicating that the patient’s RLN was not damaged during MWA

### Efficacy

**Fig. 7:**
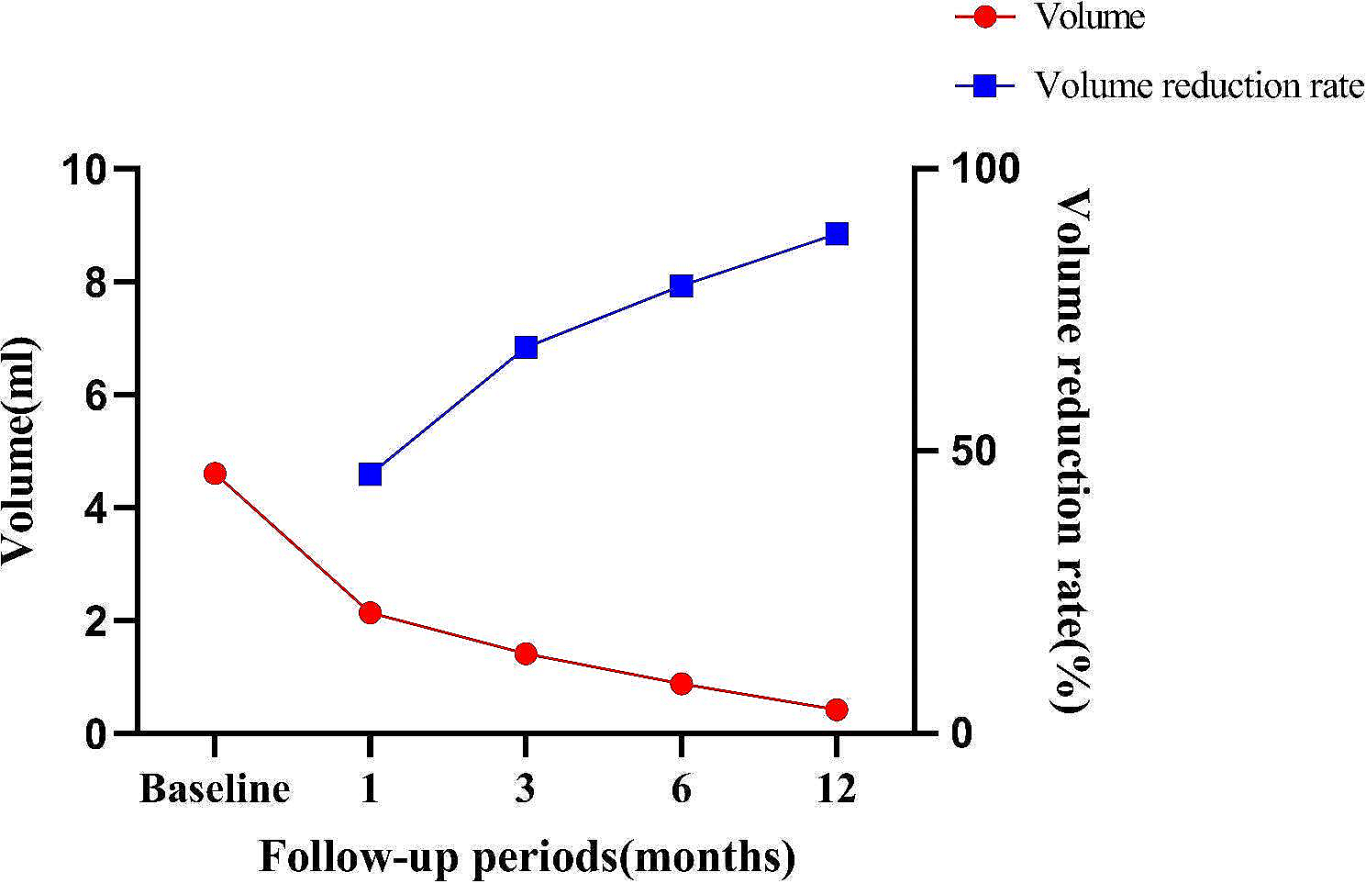
The changes in nodule volumes and the volume reduction rate after MWA were assessed at each follow-up period

All the ZTTNs were ablated successfully in this study (see two cases in Figs. 5 and 6). Patients were monitored for 12 months after undergoing MWA. The nodule volumes and VRR at each follow-up point are presented in Table 4. The median nodule volume had significantly decreased from 4.61 (2.34, 8.70) ml to 0.42 (0.15, 1.41) ml at 12months after ablation (*P* < 0.001). The VRR after MWA was 46.09 ± 25.82% after one month, 64.22 ± 22.37% after three months, 75.72 ± 18.12% after six months, and 84.36 ± 13.87% after 12 months. No nodules regrew throughout the 12-month follow-up period. Figure 7 illustrates the nodule volume and VRR at each follow-up stage following MWA.

**Table 4:** Changes in nodule volume and reduction rate after MWA at each follow-up point

Time	Volume				VRR		*P*
	Median	Range	Percentiles		Mean ± SD(%/)	Range	
			25	75			
Baseline	4.61	0.11–31.23	2.34	8.70	/	/	/
1 month	2.14	0.08–14.44	0.89	3.97	46.09 ± 25.82	2.03–95.43	*P*<0.001
3 months	1.41	0.03–11.05	0.41	2.92	64.22 ± 22.37	13.45–96.72	*P*<0.001
6 months	0.88	0.01–10.55	0.25	2.27	75.72 ± 18.12	27.40-99.32	*P*<0.001
12 months	0.42	0.01–7.14	0.15	1.41	84.36 ± 13.87	41.67–99.39	*P*<0.001

### Safety

There were no indications of ongoing bleeding, infection, recurrent laryngeal nerve injury, parathyroid gland injury, or skin scald observed in any of the patients. Additionally, none of the patients experienced dyspnea, hemorrhages/ hematomas, infections, or skin scorching at the puncture sites following MWA. Moreover, laryngeal ultrasonography showed normal vocal fold mobility for all patients pre- and post-MWA. Before MWA, the overall grade, roughness, and breathiness of the voice were moderate in 4 patients and mild in 7 patients. Eight patients experienced hoarseness after MWA. Half an hour after MWA, the overall grade, roughness, and breathiness of the voice were normal in 4 patients, while 2 patients were normal one week after MWA and 2 patients were normal two months after MWA.

## Discussion

To our knowledge, this is the first study that utilizes ultrasound to visualize RLN and ZTTN, and to lower the risk of RLN injury during MWA. Zuckerkandl’s tubercle (ZT) is a protuberance that arises from the posterior border of thyroid lobes and was first described by Emil Zuckerkandl in 1902 [22–24]. The RLN is located posterior to the ZT at the point where it enters the larynx [25]. When ZT is combined with thyroid nodules, known as ZTTN, the proximity of the thyroid nodules to the RLN increases, making it a challenging and risky procedure to ablate the thyroid nodules.

In this study, we employed high-frequency ultrasound to visualize the RLN and ZTTN prior to ablation. In high-frequency ultrasound imaging, the RLN appears as a honeycomb or reticular pattern, with roughly 2 to 8 hypoechoic rounded fascicles surrounded by hyperechoic epineurium. The nerve interior displays a striated pattern with several parallel echogenic lines on longitudinal scans. However, detecting tiny nerves using ultrasound can be challenging due to the intricacy of distinguishing minute anatomical characteristics, and this may depend on the operator and involve a longer learning curve [26–29]. Nevertheless, the liquid contrast of the barrier between the RLN and ZT during US-guided MWA procedure made it easier to identify the RLN. He et al. reported that the RLN had a thickness of 1.8 to 2.5 mm and a breadth that varied from 2 to 2.7 mm [30]. In our study, we found that the RLN had a thickness of about 2.510 ± 0.349 mm and a width of about 2.768 ± 0.399 mm. Additionally, we observed that the majority of ZTTN grades were categorized as grade 2, with the distance from the farthest outside of ZTTN to the outer edge of thyroid ranging between 6.0 and 10.0 mm. Furthermore, the position relationship between ZTTN and RLN was mainly classified as type A, where the RLN is situated at the back of ZTTN. These results are consistent with previous studies that have confirmed most RLN run posterior and medial to the ZT [23, 31–33]. In this content, even if the nodule is small, it is easy to cause RLN jamming or adhesion. Indeed, the pre-ablation visualization of RLN and ZTTN is crucially important given the potential risks associated with injury to the RLN during ablation.

In total, we included 113 ZTTNs in this study. We have successfully visualized all of them and the related RLNs using ultrasound. The information, including the size, shape and the position relationship between the ZTTN and RLN, which have been confirmed on 10 patients with RLN invasion in their surgery, guided the ablation process. Given the ZT’s unique anatomical structure, even small ZTTN can easily adhere to the RLN due to inflammation and other reasons. Therefore, separating nerve adhesions with physiological saline during ablation can be an effective method. Guo et al. demonstrated that ultrasound-guided corticosteroid injection is also a valuable approach to treating carpal tunnel syndrome [34]. Our study confirmed that the adherent RLN could be separated by physiological saline with a 100% success rate. Before ablation, 99 ZTTNs were separated from RLNs after hydrodissection, while the remaining 4 required a needle to be used under ultrasound guidance due to severe adhesion. After water isolation, the distance between RLN and ZTTN significantly increased to approximately 2-6 mm. To prevent nerve damage, it is essential to maintain a distance of at least 5 mm through real-time dynamic ultrasound observation during ablation, as suggested by numerous researchers [18, 35–38]. There are also studies showing that leverage pry-off method is a simple and effective method for preventing thermal injury complications during MWA of benign thyroid nodules [39]. During the MWA, we have combined above two methods to prevent thermal damage. This involved injecting physiological saline solution, utilizing the leverage pry-off method to elevate the nodule, and increasing the distance between the RLN and the nodule. Simultaneously, the nodule was successfully ablated. Meanwhile, as the anatomy of the RLN varies, with some nerves being thin and others having branches, it is crucial to always identify the course of the RLN and pay attention to the thick branches that may adhere to nodules and potentially result in nerve damage after ablation.

As a result, the study successfully ablated all ZTTNs, with patients being monitored for 12 months following MWA. The median nodule volume significantly decreased from 4.61 ml to 0.42 ml at 12 months after ablation, and the VRR increased over time, reaching 84.36% after 12 months. No nodules regrew during the 12-month follow-up period. The safety of this procedure was confirmed as well, with no complications reported. In addition, 11 patients experienced hoarseness due to RLN entrapment before ablation, but the voice of 7 patients recovered immediately after separation of the RLN and ZTTN during MWA. After MWA, two of the remaining four patients recovered to normal after one week, while the remaining two patients recovered after two months.

The present study is subject to some limitations. First, it is a small, single-center study that only includes benign nodules. Second, it still requires a long learning curve to distinguish the RLN. Therefore, a larger, multicenter study with prospective evaluation is necessary in the future to validate these findings.

In conclusion, the RLN is closely related to ZTTN and mainly located at the back of it. Hydrodissection during MWA can separate the RLN from ZTTN. US-guided MWA is a safe and efficient treatment for ZTTN. Accurate identification of the RLN during MWA is crucial to protect the nerve and prevent nerve damage.

## Data Availability

The datasets used and/or analysed during the current study available from the corresponding author on reasonable request.

## Abbreviations

CEUS: Contrast-Enhanced Ultrasound
FNA: Fine-needle aspirations
LA: Laser ablation
MWA: Microwave ablation
RFA: Radiofrequency ablation
RLN: Recurrent laryngeal nerve
US: Ultrasound
VRR: Volume reduction rates
ZTTN: Thyroid nodules at Zuckerkandl tubercle
